# Reduced Hippocampal Dendritic Spine Density and BDNF Expression following Acute Postnatal Exposure to Di(2-Ethylhexyl) Phthalate in Male Long Evans Rats

**DOI:** 10.1371/journal.pone.0109522

**Published:** 2014-10-08

**Authors:** Catherine A. Smith, Matthew R. Holahan

**Affiliations:** Department of Neuroscience, Carleton University, Ottawa, Canada; Brock University, Canada

## Abstract

Early developmental exposure to di(2-ethylhexyl) phthalate (DEHP) has been linked to a variety of neurodevelopmental changes, particularly in rodents. The primary goal of this work was to establish whether acute postnatal exposure to a low dose of DEHP would alter hippocampal dendritic morphology and BDNF and caspase-3 mRNA expression in male and female Long Evans rats. Treatment with DEHP in male rats led to a reduction in spine density on basal and apical dendrites of neurons in the CA3 dorsal hippocampal region compared to vehicle-treated male controls. Dorsal hippocampal BDNF mRNA expression was also down-regulated in male rats exposed to DEHP. No differences in hippocampal spine density or BDNF mRNA expression were observed in female rats treated with DEHP compared to controls. DEHP treatment did not affect hippocampal caspase-3 mRNA expression in male or female rats. These results suggest a gender-specific vulnerability to early developmental DEHP exposure in male rats whereby postnatal DEHP exposure may interfere with normal synaptogenesis and connectivity in the hippocampus. Decreased expression of BDNF mRNA may represent a molecular mechanism underlying the reduction in dendritic spine density observed in hippocampal CA3 neurons. These findings provide initial evidence for a link between developmental exposure to DEHP, reduced levels of BDNF and hippocampal atrophy in male rats.

## Introduction

Phthalates are synthetically derived chemicals widely used in many common consumer plastic products [1]. They are not chemically bound to plastic polymers and are easily released into the environment [2]–[3]. Estimates of exposure to phthalates are higher in infants and children compared to adults [4]–[5] highlighting the importance of examining the effects of phthalate exposure on developing organisms. Animal toxicity studies have shown severe defects in male reproductive organs following early life exposure to phthalates in animals and humans, such as undescended testes [1], [6]–[8]. Male lab animals also show a permanent feminization of the reproductive system, including the retention of nipples and decreased anogenital distance [7]. Reduced anogential distance has also been observed in human studies, with an inverse relationship between maternal exposure to phthalates and anogential distance in male infants [8].

Extensive growth and re-organization of neurocircuitry occurs during postnatal development, leaving the brain highly susceptible to environmental insults at this time [9]. The impact of early life exposure to phthalates on brain development, however, has not been widely studied. One brain region, the hippocampus, has shown a particular sensitivity to early developmental exposure to phthalates [10]–[11]. Specifically, the dorsal hippocampus undergoes a period of intense growth and dendritic/axonal expansion during early postnatal development in rats [12]–[14]. Exposure to phthalates during these important hippocampal developmental periods may increase risk of disruption to developmental processes producing long-lasting changes in hippocampal neurocircuitry.

Experimental investigations exploring structural-based alterations in hippocampal connectivity in the rat following early developmental phthalate treatment are limited. Systemic treatment with 10 mg/kg di(2-ethylhexyl) phthalate (DEHP) during postnatal development led to a marked reduction in synaptophysin staining (a protein critically involved in synapse formation and maintenance) in the CA3 stratum oriens dorsal hippocampal region of male rats, indicating decreased axonal innervation to this region [10]. A reduction in synaptophysin protein expression, along with disruptions in synaptic ultrastructure (such as loss of synapses and a wider synaptic gap), were observed in the hippocampus of male and female rats following perinatal dibutal phthalate (DBP) exposure [11]. These studies indicate early developmental exposure to phthalates may interfere with proper synaptic development in the dorsal hippocampus. The formation and maintenance of synaptic connections in developing organisms is critical for proper dendritic arborisation, and the failure to form these connections during development can disrupt dendritic outgrowth [15]–[17].

Connectivity-based changes in the brain are likely mediated through coordinated increments in growth factors and decrements in anti-growth factors. Brain-derived neurotrophic factor (BDNF) is a growth factor which plays an important role in neuronal and synaptic development, including the stimulation of dendritic and axonal growth in hippocampal neurons [18]–[20]. The exogenous application of BDNF increased axonal growth in cultured dentate granule cells [21]. Conversely, decreased hippocampal BDNF *in vitro* and *in vivo* may directly reduce dendritic spine outgrowth in hippocampal neurons [22]–[23]. The effect of early developmental phthalate exposure on BDNF expression in the brain has not been thoroughly investigated. Only two studies have examined the relationship between phthalate treatment and BDNF levels, and have produced contradictory findings. Perinatal treatment with 500 mg/kg of DBP decreased BDNF expression in the hippocampus [11]. Conversely, perinatal treatment with a higher dose of DBP (675 mg/kg) increased levels of hippocampal BDNF expression [24]. These studies suggest a possible role for phthalates in regulating hippocampal BDNF expression.

BDNF also provides support to neurons to promote their survival [25]. The protective effect of BDNF against cell apoptosis is largely through the suppression of caspase-3 activity [25]. *In vitro* treatment with low doses of DEHP enhanced caspase-3 protein expression and reduced the viability of Neuro-2a, MVLN, and AGS cell lines [26]–[28]. Exposure to mono(2-ethylhexyl) phthalate (MEHP; the primary metabolite of DEHP) *in vitro* also reduced U937 cell viability, increased caspase-3 and BAX (a pro-apoptotic protein) expression, and decreased Bcl-2 (an anti-apoptotic protein) expression [29]. MEHP treatment has also been found to initiate apoptosis in the testes of rats and mice via increased caspase-3 expression, oxidative stress, and Fas-signalling activation [30]–[33].

In the brain, postnatal exposure to low doses of DEHP has been shown to reduce the density of mature and immature dorsal hippocampal neurons in the male rat, suggesting heightened cell apoptosis in the hippocampus [10]. Perinatal DBP exposure stimulated caspase-3 activity in rats and significantly reduced the density of mature hippocampal neurons [11]. Other apoptotic markers, including TUNEL and Annexin V-propidium iodide, were also up-regulated in the hippocampus; further supporting the idea of enhanced cell apoptosis following early developmental exposure to phthalates [11].

The primary goal of this work was to further characterize the impact of postnatal phthalate exposure on dorsal hippocampal development in juvenile rats. Hippocampal dendritic morphology (branching, spine density) and mRNA expression of BDNF and caspase-3 were evaluated in male and female juvenile rats following repeated postnatal exposure to 10 mg/kg DEHP. It was expected that DEHP exposure would reduce dendritic branching and spine density, and increase BDNF and caspase-3 mRNA expression in the hippocampus. A secondary goal was to evaluate DEHP mediated effects across gender. Previous work has suggested that male rats may be more vulnerable to early developmental DEHP exposure [10] and thus, it was expected that these alterations in hippocampal dendritic morphology and mRNA expression would be more pronounced in males than in females.

## Materials and Method

### Ethical Statement

All experiments were conducted at Carleton University and approved by the Carleton University Animal Care and Use Committee as per guidelines established by the Canadian Council on Animal Care (Protocol #: P10–37). The number of animals used was kept to a minimum (n = 5/group) and safeguards were in place to ensure minimal suffering.

### Animals

Four untimed pregnant female Long Evans rats (approximately 13 days gestation) were purchased from Charles River Laboratories (St. Constant, Québec). The pregnant females were singly-housed in polycarbonite cages (48×26×20) within a temperature controlled environment. The day the pups were born was recorded as postnatal day (PND) 0. Pups (n = 20 males; n = 20 females) were weaned on PND22 and group-housed, with males and females in separate cages. Mean weight on PND16, PND22, and PND26 was 29.0 g, 47.2 g, and 65.6 g for male rats, respectively. Mean weight on PND16, PND22, and PND26 was 28.2 g, 46.0 g, and 62.9 g for female rats. All rats were on a 12 hour light-dark cycle (lights on at 8:00 a.m.) with *ad libitum* access to food (Purina rat chow) and tap water.

### DEHP Injections

DEHP (Sigma-Aldrich; St. Louis, MO, USA) or vehicle (corn oil) was injected intraperitoneal (i.p.) into awake rats daily from PND16 to PND22 inclusive (n = 10 males; n = 10 females). Each rat was injected in the late morning (between 10:30 and 11:00 AM) and was returned to their home cage with their mother following the injection. Rats were randomly assigned to treatment (DEHP) and control (vehicle) groups counterbalanced across all litters. The 10 mg/kg DEHP solution was prepared fresh using DEHP (1000 mg/kg) and corn oil immediately before each injection. This dose was chosen based on previous neurotoxicology findings showing abnormal hippocampal development in male Long Evans rats following acute postnatal treatment with 10 mg/kg DEHP [10] and reproductive toxicology findings that show doses as low as 10 mg/kg lead to decreased testosterone production [34]. Rats (n = 10 males; n = 10 females) receiving vehicle injections were injected with corn oil.

### Golgi-Cox Impregnation Method

#### Tissue Processing

Rats (n = 5 male DEHP; n = 5 female DEHP; n = 5 vehicle male; n = 5 vehicle female) were euthanized on PND26. In Long Evans rats, the axonal terminal fields in the hippocampal CA3 region reflect the connectivity patterns observed in adulthood by PND24 [12]. Rats were euthanized on PND26 (4 days after the last treatment) to ensure the hippocampus had fully developed prior to morphological investigation. Rats were euthanized via transcardial perfusion with 0.9% physiological saline following overdose of Dorminal (0.2 ml of 50 mg/kg). Brains were extracted and prepared for Golgi impregnation using the Golgi-Cox technique. Immediately after extraction, brains were steeped in potassium dichromate, mercuric chloride, and potassium chromate solution (Golgi fix solution) for 4 days. Following this period, brains were washed 3 times in distilled H_2_O (DH_2_O): 4 hours, 3 hours and overnight, respectively. Brains were then cryoprotected in a graduated sucrose sequence: 10% sucrose for 8 hours, 20% sucrose overnight and 30% sucrose for a minimum of 4 days. Dorsal hippocampal sections (200 µm) were obtained using a Vibratome and mounted onto gelatinized slides. The mounted sections were placed in a humidified, dark plastic box for 24 hours. Each slide with mounted sections was rinsed in DH_2_O for 1 minute and submerged in 28% Ammonium Hydroxide for 40 minutes. Sections were rinsed in DH_2_O for 1 min and submerged in Kodak film fix A (diluted 1∶1 with DH_2_O) for 40 minutes. They were then rinsed two more times in DH_2_O for 1 minute each before being immersed in 50%, 70% and 95% ethanol for 1 min each. From this point on, all ethanol and Clearene solutions were desiccated with type 3A molecular sieve, 1/16” pellets. Sections were immersed three times in 100% ethanol for 5 minutes each then submerged in a 33% ethanol, 33% Clearene and 33% chloroform solution for 10 minutes. Sections were immersed two times in a Clearene solution for 15 minutes each before the slides were coverslipped with Permount mounting medium. The slides were then placed in a desiccated box for 4 days before morphological analysis.

#### Morphological analysis

Individual neurons were reconstructed at 100× magnification on an Olympus BX51 microscope using Neurolucida software (MBF Bioscience Inc., Williston, VT). Neurons were selected at random from the dorsal hippocampal for each hippocampal region: DG, CA1 and CA3. Approximate bregma level −3.14 to −3.60 mm. A total of 3–5 neurons were traced for each hippocampal region per rat and were averaged across each region per rat. Neurons that were reconstructed were consistently impregnated and did not overlap with neighbouring cells. For all neurons, the cell body, apical and basal dendrites, and visible dendritic spines were reconstructed. Measurements recorded from each neuronal tracing included cell body area, the number of dendritic branch points, and dendritic spine density for both basal and apical dendrites. An experimenter who was blind to group assignment carried out all analyses.

### Reverse Transcription-Quantitative Polymerase Chain Reaction (qPCR) Analysis

A second group of rats (n = 5 male DEHP; n = 5 female DEHP; n = 5 vehicle male; n = 5 vehicle female) were euthanized on PND26 by overdose (Dorminal; 0.2 ml of 50 mg/kg). Brains were extracted and the dorsal hippocampus was collected from each rat. Tissue was placed in tubes on dry ice and stored at −80°C for later quantification of mRNA expression of genes of interest: BDNF and caspase-3, and a housekeeping gene (GAPDH).

Tissue was homogenized in Trizol and RNA was extracted as per instructions from commercially available PureLink RNA Micro Scale Kit (Thermo Fisher Scientific Inc., Catalog #: 12183016). The RNA was then reverse-transcribed into cDNA using SuperScript II reverse transcriptase (Invitrogen, Burlington, ON, Canada). The resulting cDNA samples were analyzed via qPCR using a SYBR green detection protocol (Bio-Rad, CA, USA) and a MyiQ2 RT-PCR Detection System (Bio-Rad, CA, USA). All primer pairs generated 100–200 base pair amplicons and had a minimum efficiency of 90%. The cycle threshold (Ct) of the housekeeping gene GAPDH was subtracted from the Ct of each gene of interest (ΔCt) to normalize BDNF and caspase-3 expression levels. ΔCt values were converted to mRNA fold changes using the 2^−ΔΔCT^ method [35]–[36]. Primer sequences used: BDNF forward: GGACATATCCATGACCAGAAAGAAA, reverse: GCAACAAACCACAACATTATCGAG; caspase-3 forward: ATTGAGACAGACAGTGGAAC, reverse: GAGGAATAGTAACCGGGTG; GAPDH forward: GCCATCAACGACCCCTTCAT, reverse: CCGCCTGCTTCACCACCTTC.

### Statistical Analyses

Morphological data from reconstructed neurons were analyzed using fixed factor analysis of variance (ANOVA) with gender (male and female) and treatment (DEHP and vehicle) as the fixed factors. Independent variables included cell body area, dendritic branching, and dendritic spine density in the DG, CA1, and CA3. Reverse transcription qPCR data were analyzed using fixed factor ANOVAs with gender (male and female) and treatment (DEHP and vehicle) as the fixed factors. P-values of less than 0.05 were considered statistically significant. Simple main effect follow-up analyses and pairwise comparisons were conducted using a Bonferroni correction to control from Type I error.

## Results

### No Effect of DEHP Treatment on Cell Body Size or Dendritic Branching

The effect of early developmental exposure to DEHP on cell body area and dendritic branching in the DG, CA1, and CA3 regions of the dorsal hippocampus was evaluated in male and female juvenile rats (n = 5/per group. No differences were observed in the total area of the cell bodies located in the DG, CA1 or CA3 between DEHP- and vehicle-treated rats of either gender (*p*>0.05). The total number of branches on apical and basal dendrites of CA1 and CA3 neurons, and on basal dendrites of DG granule cells following DEHP exposure was not significantly different from vehicle controls in male or female rats (*p*>0.05).

### DEHP Exposure Reduced Dendritic Spine Density on CA3 Neurons in Male Rats

**Figure 1:**
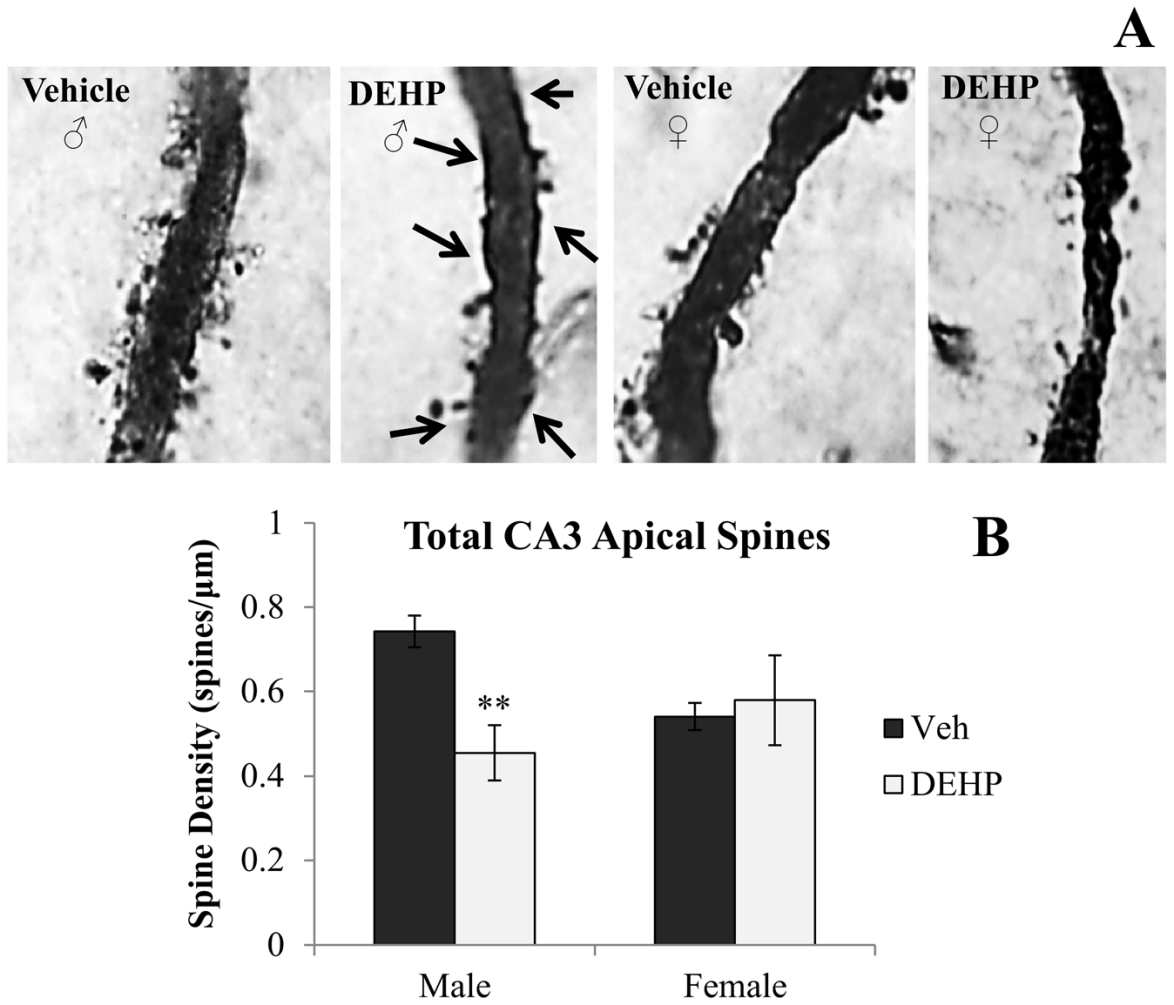
Spine density on apical dendrites of dorsal CA3 hippocampal neurons. (A) Representative photomicrographs of spine density on second order apical dendritic branches of CA3 neurons (100x). Quantification of the total number of basal dendritic spines per µm (B) in male and female rats exposed to di(2-ethylhexyl) phthalate (DEHP) compared to controls (Veh). Male rats exposed to DEHP showed a significant reduction in spine density on CA3 apical dendrites compared to male controls. Arrows in the DEHP-male image show regions of reduced dendritic spine density. There was no significant effect of DEHP treatment on CA3 apical spine density in female rats compared to controls. Error bars represent standard error of the mean. ** = *p*<0.01.

**Figure 2:**
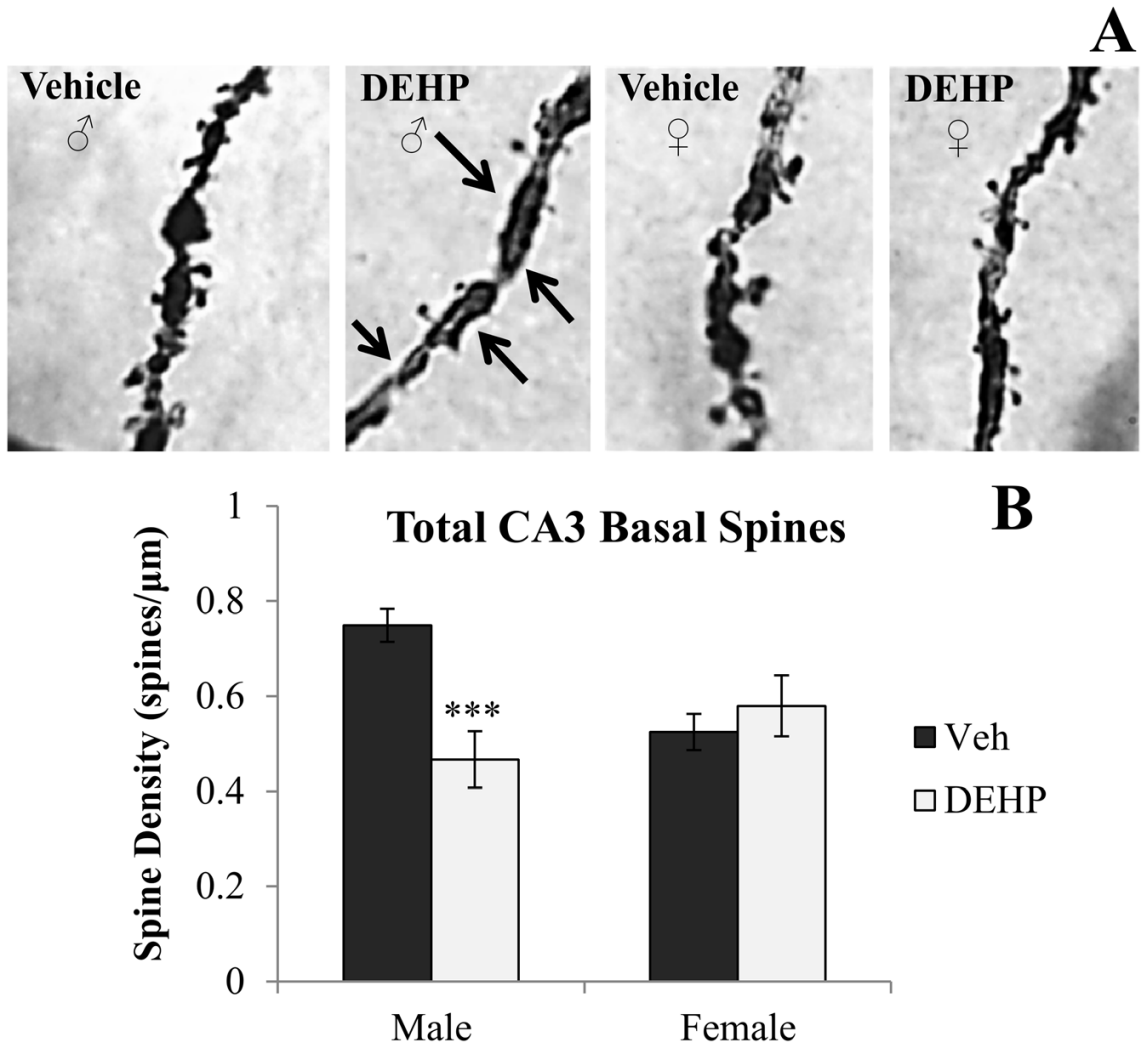
Spine density on basal dendrites of dorsal CA3 hippocampal neurons. (A) Representative photomicrographs of spine density on fourth order basal dendritic branches of CA3 neurons (100x). Quantification of the total number of basal dendritic spines per µm (B) in male and female rats exposed to di(2-ethylhexyl) phthalate (DEHP) compared to controls (Veh). Male rats exposed to DEHP showed a significant reduction in spine density on CA3 basal dendrites compared to controls. Arrows in the DEHP-male image show regions of reduced dendritic spine density. There was no significant effect of DEHP treatment on CA3 basal spine density in female rats compared to controls. Error bars represent standard error of the mean. *** = *p*<0.001.

**Figure 3:**
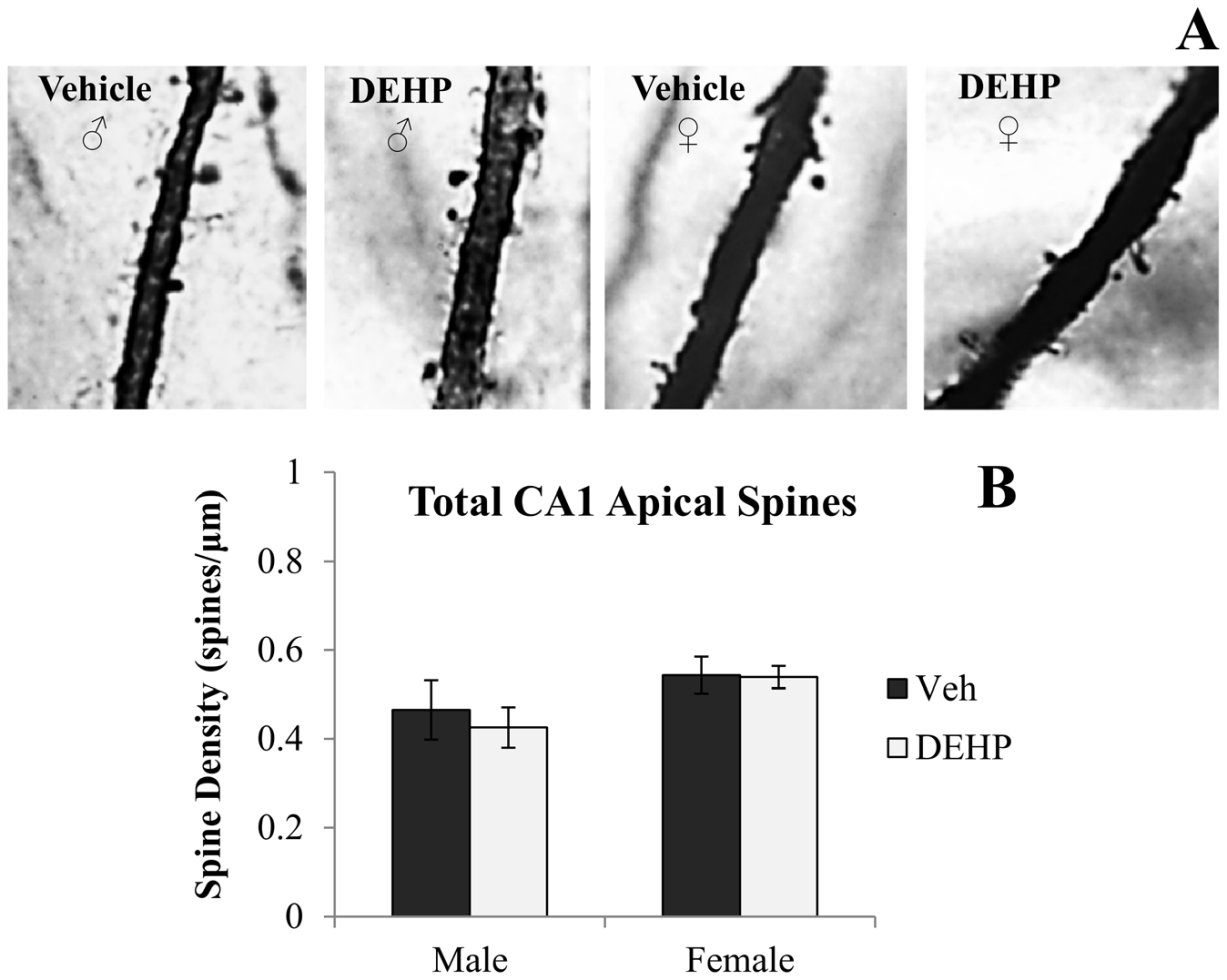
Spine density on apical dendrites of dorsal CA1 hippocampal neurons. (A) Representative photomicrographs of spine density on second order apical dendritic branches of CA1 neurons (100x). Quantification of the total number of basal dendritic spines per µm (B) in male and female rats exposed to di(2-ethylhexyl) phthalate (DEHP) compared to controls (Veh). There were no differences in CA1 apical dendritic spine density in male or female rats exposed to DEHP compared to controls. Error bars represent standard error of the mean.

**Figure 4:**
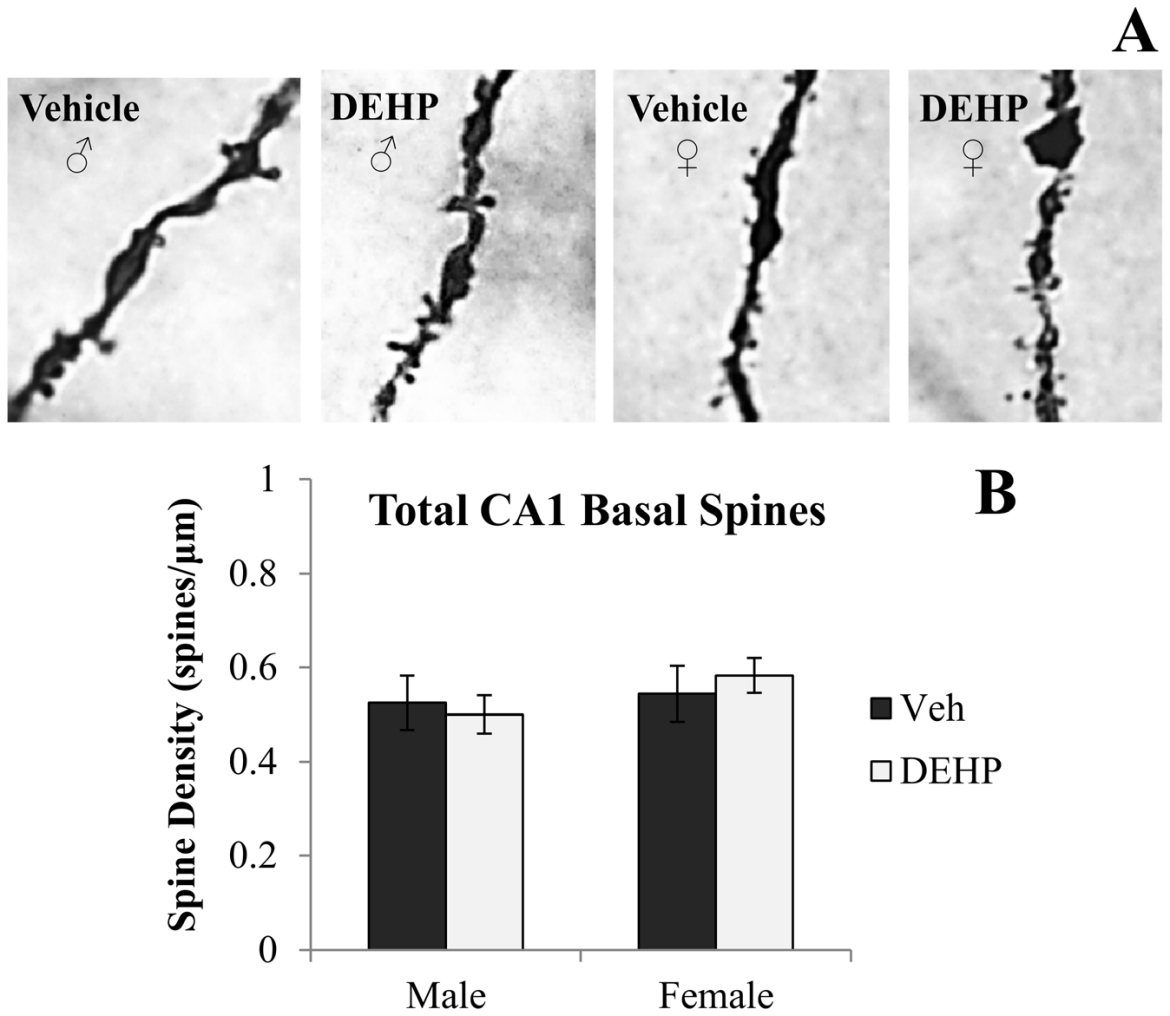
Spine density on basal dendrites of dorsal CA1 hippocampal neurons. (A) Representative photomicrographs of spine density on fourth order basal dendritic branches of CA1 neurons (100x). Quantification of the total number of basal dendritic spines per µm (B) in male and female rats exposed to di(2-ethylhexyl) phthalate (DEHP) compared to controls (Veh). There were no differences in CA1 basal dendritic spine density in male or female rats exposed to DEHP compared to controls. DEHP: di(2-ethylhexyl) phthalate. Error bars represent standard error of the mean.

**Figure 5:**
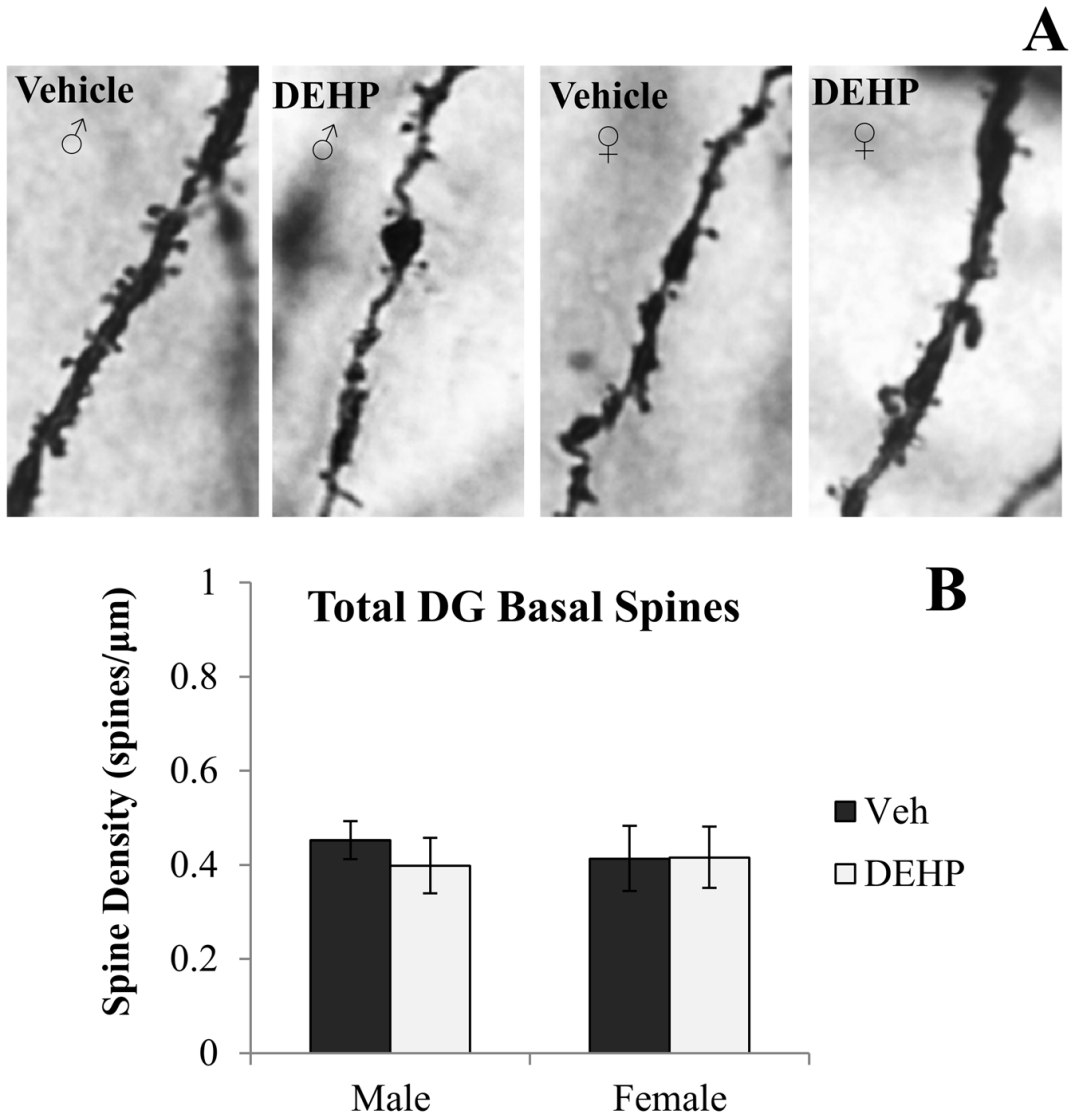
Spine density on basal dendrites of dorsal dentate gyrus hippocampal granule cells. (A) Representative photomicrographs of spine density on fourth order basal dendritic branches of dentate gyrus granule cells (100x). Quantification of the total number of basal dendritic spines per µm (B) in male and female rats exposed to di(2-ethylhexyl) phthalate (DEHP) compared to controls (Veh). There were no differences in DG basal dendritic spine density in male or female rats exposed to DEHP compared to controls. Error bars represent standard error of the mean.

Figure 1A and 2A shows representative photomicrographs of spine density on apical and basal dendrites of CA3 neurons, respectively. Analyses revealed significant gender × treatment interactions of spine density on both apical (*F*(1,16)  = 5.90, *p*<0.05; Figure 1B) and basal dendrites (*F*(1,16)  = 11.03, *p*<0.01; Figure 2B). Simple main effects and pairwise comparisons revealed reductions in CA3 apical and basal spine density between DEHP-treated male rats and male controls (*F*(1,16)  = 9.14, *p*<0.01; *F*(1,16)  = 15.46, *p*<0.001; respectively). DEHP treatment did not alter dendritic spine density in apical or basal dendrites of CA3 neurons in female rats (*F*(1,16)  = 0.17, *p* = 0.69; *F*(1,16)  = 0.59, *p* = 0.46; respectively). Figures 3A, 4A, and 5A show representative photomicrographs of spine density on apical and basal dendrites of CA1 neurons, and on basal dendrites on DG granule cells, respectively. No differences in spine density on basal or apical dendrites of CA1 neurons or basal dendrites on DG granule cells were found between DEHP-treated male and female rats when compared to controls of the same gender (*p*>0.05; Figure 3B; 4B; 5B).

### DEHP Exposure Reduced Hippocampal BDNF mRNA Expression in Males

**Figure 6:**
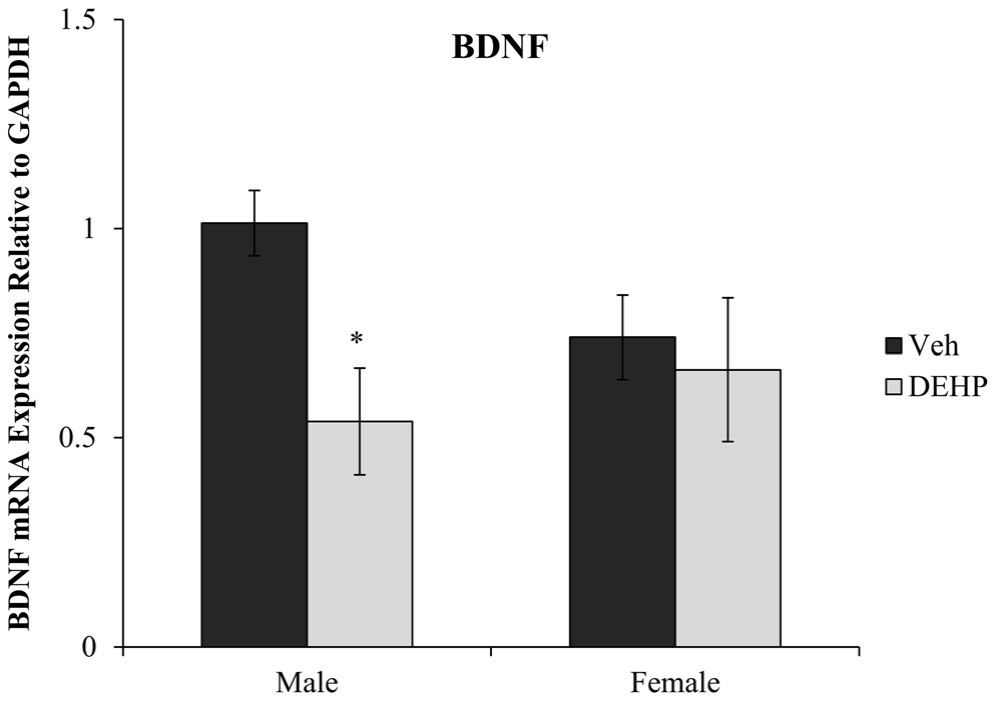
BDNF mRNA expression the dorsal hippocampus. Quantification of BDNF mRNA expression relative to housekeeping gene (GAPDH) in male and female rats exposed to di(2-ethylhexyl) phthalate (DEHP) compared to controls (Veh). Male rats exposed to DEHP showed a significant reduction in BDNF mRNA expression compared to controls. There was no significant effect of DEHP treatment on BDNF mRNA expression in female rats compared to controls. Error bars represent standard error of the mean. * = p<0.05.

**Figure 7:**
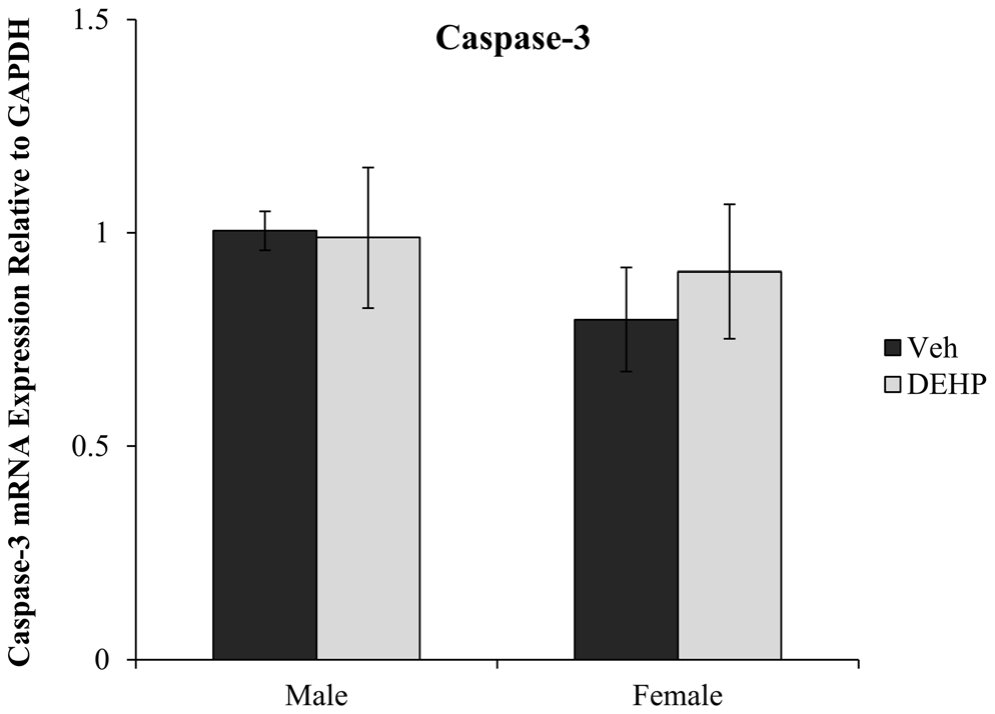
Caspase-3 mRNA expression the dorsal hippocampus. Quantification of caspase-3 mRNA expression relative to housekeeping gene (GAPDH) in male and female rats exposed to di(2-ethylhexyl) phthalate (DEHP) compared to controls (Veh). There were no differences in caspase-3 mRNA expression in male or female rats exposed to DEHP compared to controls. Error bars represent standard error of the mean.

There was a main effect of DEHP treatment (*F*(1,16)  = 4.90, *p*<0.05), but no effect of gender on BDNF mRNA expression in the hippocampus of juvenile rats (Figure 6). This reduction in BDNF transcripts was restricted to male rats, with a 53.2% reduction in BDNF mRNA expression in DEHP-treated male rats compared to male controls (*F*(1,16)  = 7.25, *p*<0.05). No alterations in BDNF mRNA expression were found between DEHP-treated and control female rats, indicating male rats may be more sensitive to postnatal DEHP exposure (*F*(1,16)  = 0.19, *p* = 0.67). No differences were observed in casapase-3 mRNA expression between DEHP-treated male and female rats compared to controls of the same gender (*p*>0.05; Figure 7).

## Discussion

The present study evaluated alterations in dorsal hippocampal dendritic morphology, and BDNF and caspase-3 mRNA expression following exposure to a low dose of DEHP during early postnatal development in male and female rats. DEHP exposure did not alter cell body size or the number of dendritic branches on DG, CA1 or CA3 neurons in male or female rats. Structural changes in dendritic complexity were specific to reductions in spine density on CA3 apical and basal dendrites in DEHP-treated male rats compared to controls. No differences in spine density were observed in male rats in the DG or the CA1, and no differences in spine density were observed in any dorsal hippocampal region in female rats.

DEHP-induced reductions in CA3 spine density in male rats may have profound consequences for hippocampal connectivity. Synaptic innervation of the CA3 dorsal hippocampal region occurs through three distinct pathways: the perforant pathway, the mossy fiber pathway and the recurrent collaterals within the CA3 itself [37]–[38]. In the perforant pathway, information is relayed through projections from the entorhinal cortex to distal CA3 apical dendrites located in the stratum lacunosum-moleculare hippocampal layer. Mossy fiber synaptic inputs (from DG granule cells) terminate on more proximal CA3 apical dendrites in the stratum lucidum, whereas recurrent collaterals from CA3 neurons project back to the CA3 region, synapsing on basal dendrites in the stratum oriens, and mid-apical dendrites in the stratum radiatum [37]–[38]. In the present study, a reduction in spine density was observed throughout the entire CA3 dendritic tree, including CA3 basal dendrites and proximal, mid, and distal CA3 apical dendrites following DEHP treatment in male rats. These findings suggest that early developmental exposure to DEHP may lead to a widespread reduction in synaptic connections to the CA3 from multiple brain regions in male rats, including the DG, the entorhinal cortex, and the CA3.

Structural changes at synapses, including dendritic spine formation, have a direct influence on synaptic function [39] and thus, it is conceivable that this reduction in dendritic spine density in DEHP-treated male rats may also lead to a corresponding decrease in synaptic efficacy. In a recent experiment, the effect of perinatal DBP exposure on synaptic function of rat CA1 neurons was investigated [11]. DBP treatment reduced the slope and the amplitude of field excitatory postsynaptic potentials in CA1 neurons, indicating a decrease in synaptic function [11]. Electrophysiological experiments evaluating synaptic function in DG, CA1 and CA3 hippocampal neurons following DEHP exposure are recommended. It is expected that a similar decline in synaptic efficacy would be observed in the CA3 due to the decrease in spine density.

The reduction in spine density on CA3 neurons in male DEHP-treated rats parallels our previous findings indicating that postnatal exposure to DEHP decreased axonal innervation in the dorsal CA3 distal stratum oriens of male rats [10]. Abnormal hippocampal synaptic ultrastructure, including the loss of synapses, was also found following perinatal DBP treatment in male and female rats [11]. These findings highlight a pattern of abnormal hippocampal connectivity and development following phthalate treatment, establishing a link between early developmental exposure to phthalates and considerable reduction in hippocampal synaptic complexity.

No differences in dendritic spine density were observed in the DG or CA1 of DEHP-treated male or female rats compared to their respective controls. This finding is partially consistent with previous studies that revealed significant changes in hippocampal morphology in the DG and CA3, but not the CA1 [10]. The rats in the present study were exposed to DEHP between PND16–22, a period of significant dorsal CA3 hippocampal development [12]. The peak developmental time period for the dorsal CA1 hippocampal region begins at birth and continues until PND7 [13]. For the DG, the peak developmental time period is from PND4 to PND11 [14]. Since the CA1and DG region had likely already fully developed at the time of DEHP exposure (PND16–22), perhaps this region was less sensitive to DEHP exposure and no significant alterations in hippocampal dendritic morphology were observed.

Dorsal hippocampal BDNF mRNA expression was also down-regulated by approximately 50% in male rats exposed to DEHP (10 mg/kg). No differences in BDNF expression were observed in female rats. The down-regulation of hippocampal BDNF mRNA expression in DEHP-treated male rats may represent a molecular mechanism mediating the reduction in dendritic spine density in DG and CA3 neurons. BDNF is important for dendritic outgrowth and the formation of new synaptic connections [18]–[20]. These findings highlight the importance of investigating the probable connection between DEHP-induced down-regulation of BDNF (and other genes involved in dendritic/axonal growth) and structural-based alterations in hippocampal morphology.

BDNF also plays a critical role in regulating cell apoptosis via caspase-3 suppression [25]. DEHP-treatment did not change expression levels of hippocampal caspase-3 mRNA expression in male or female rats despite the significant down-regulation of hippocampal BDNF expression. This finding was unexpected as previous studies had reported phthalate-induced reductions in hippocampal cell density and the up-regulation of caspase-3 activity [10]–[11]. In previous work from our lab using the same DEHP exposure procedure, decreased dorsal CA3 hippocampal cell densities were reported in DEHP-treated male rats on PND26 [10]. It is possible that caspase-3 activity may have been up-regulated during the DEHP treatment days (PND16–22) leading to reduced CA3 hippocampal cell density. Once DEHP treatment ended on PND22, caspase-3 activity would have returned to baseline levels and thus, no differences in caspase-3 mRNA expression would have been detected when assessed on PND26.

This hypothesis is consistent with a previous study that showed increased caspase-3 expression only on days during which phthalate treatment occurred [11]. Increased hippocampal caspase-3 expression (along with reductions in hippocampal cell density) at PND5 and PND21 were observed following exposure to DBP from gestational day 7 to PND21 in male and female rats [11]. When caspase-3 expression was measured 5 weeks after the last day of phthalate treatment, no differences in caspase-3 expression were present suggesting heightened caspase-3 activity may occur only in conjunction with simultaneous phthalate treatment. While it appears caspase-3 activity may return to normal levels once phthalate treatment is terminated, the long-term neurodevelopmental and behavioural consequences of phthalate-induced alterations in neurochemical signalling pathways, including caspase-3 and BDNF, are unknown and require further investigation.

Reductions in hippocampal dendritic spine density and BDNF mRNA expression were gender-specific. No differences in DG or CA3 dendritic spine densities or in BDNF mRNA expression were observed between DEHP- and vehicle-treated female rats, suggesting male rats may be particularly vulnerable to early developmental DEHP exposure. This finding is consistent with a previous study from our laboratory revealing alterations in dorsal hippocampal morphology following postnatal DEHP exposure in male, but not female rats [10]. The vulnerability of male rats to DEHP exposure may involve the anti-androgenic properties of DEHP. Low doses of DEHP have been shown to supress aromatase enzyme activity and reduce serum testosterone levels (similar to levels seen in females) in male rats [34], [40]–[45]. DEHP and MEHP treatment in female rats has been associated with decreased levels of estradiol and progesterone [46]–[49].

Gonadal hormones are critically involved in the organization and maintenance of synaptic connections in the brain [50]–[53]. There is evidence for sexual dimorphism in the regulation of spine synaptic density on dorsal CA1 pyramidal neurons in adult rats. There is an activational effect of estrogen and progesterone on CA1 spine synaptic density in female rats [54]–[57], but not male rats [53]. There is also an activational effect of androgens (testosterone and dihydrotestosterone) on CA1 spine synaptic density in both male and female adult rats [51]–[53]. Spine density in cultured CA3 pyramidal neurons in adult male rats is decreased following the application of estradiol [58], but increased in the presence of testosterone or dihyrdrotestosterone [59].

Estrogen and testosterone also appear to have a neuroprotective effect on cell survival. The application of estrogen to cultured hippocampal neurons from rat pups (gestational day 18) increased Bcl-xL (an anti-apoptotic protein) expression and reduced beta-amyloid-induced apoptosis [60]. *In vivo*, estrogen deprivation (by ovariectomy) in adult female rats potentiated apoptosis in DG granule cells when compared to controls [61]. Treatment with exogenous 17 β-estradiol reversed apoptosis in ovariectomized rats indicating low physiological levels of estrogens increases the susceptibility of DG granule cells to cell death [61]. There was no added benefit of elevated 17 β-estradiol treatment in rats with normal physiological levels of estrogen [61].

The application of testosterone or dihydrotestosterone in cultured hippocampal neurons from rat pups (gestational day 17–19) attenuates beta-amyloid-induced apoptosis [62]–[63]. The neuroprotective effects of testosterone and dihydrotestosterone can be blocked by preventing the phosphorylation of pro-apoptotic protein BAD [62]. Conversely, elevated levels of testosterone (above normal physiological levels) promoted apoptosis in cultured human neuroblastoma cells while elevated levels of 17 β-estradiol had no effect [64].

Together these findings suggest that abnormal levels of gonadal hormones may have profound consequences for spine growth and cell survival. Given the endocrine disruption properties of DEHP, it is possible that altered levels of these gonadal hormones may be contributing to reductions in CA3 synaptic connectivity and spine growth in male rats. This may also explain the gender-specific detriment of DEHP on the hippocampus of male rats, and suggest that altered testosterone and/or aromatase enzyme activity may be involved in DEHP-induced hippocampal dysfunction. Further investigation is required to determine whether these changes in hippocampal neurobiology in DEHP-treated male rats can be attributed to low levels of testosterone and/or the suppression of aromatase activity. Circulating levels of gonadal hormones in female rats will also need to be assessed to uncover possible explanations as to why DEHP treatment had no effect on female hippocampal neuroconnectivity.

### Conclusions

The present results revealed that exposure to DEHP between PND16-PND22 in male rats reduced spine density on basal and apical dendrites of CA3 neurons compared to vehicle-treated males. Hippocampal BDNF mRNA expression was also down-regulated in male rats exposed to DEHP and may represent a molecular mechanism underlying the reduction in dendritic spine density. No differences in hippocampal spine density or hippocampal BDNF mRNA expression were observed in female rats treated with DEHP compared to controls, suggesting male rats may be more vulnerable to early developmental DEHP exposure. DEHP treatment did not affect hippocampal caspase-3 mRNA expression in male or female rats. These data provide initial evidence between early developmental exposure to DEHP and neurodevelopmental deficits in male rats, as well as highlight the importance of elucidating molecular mechanisms of DEHP-induced neurodevelopmental dysfunction.
